# Risk assessment in the plateau pika *(Ochotona curzoniae)*: intensity of behavioral response differs with predator species

**DOI:** 10.1186/s12898-020-00309-3

**Published:** 2020-07-17

**Authors:** Wanrong Wei, Qiaoyan Zhen, Zhongmin Tang, Maria K. Oosthuizen

**Affiliations:** 1 Key Laboratory of Southwest China Wildlife Resources Conservation, College of life Sciences, China West Normal University, Nanchong, 637009 China; 2 State Key Laboratory of Grassland Agro-Ecosystems, College of Pastoral Agriculture Science and Technology, Lanzhou University, Lanzhou, 730200 China; 3 China West Normal University, Nanchong, 637009 China; 4 Gannan Grassland Workstation in Gansu Province, Hezuo, 747000 China; 5 Department of Zoology and Entomology, University of Pretoria, Private Bag X20, Hatfield, 0028 South Africa; 6 Mammal Research Institute, University of Pretoria, Hatfield, 0028 South Africa

**Keywords:** Predator–prey interactions, Plateau pika, Anti-predator behavior, FID, The hiding time, Vigilance

## Abstract

**Background:**

The ability of a prey species to assess the risk that a predator poses can have important fitness advantages for the prey species. To better understand predator–prey interactions, more species need to be observed to determine how prey behavioral responses differ in intensity when approached by different types of predators. The plateau pika (*Ochotona curzoniae*) is preyed upon by all predators occurring in its distribution area. Therefore, it is an ideal species to study anti-predator behavior. In this study, we investigated the intensity of anti-predator behavior of pikas in response to visual cues by using four predator species models in Maqu County on the eastern Qinghai-Tibetan Plateau.

**Results:**

The behavioral response metrics, such as Flight Initiation Distance (FID), the hiding time and the percentage of vigilance were significantly different when exposed to a Tibetan fox, a wolf, a Saker falcon and a large-billed crow, respectively. Pikas showed a stronger response to Saker falcons compared to any of the other predators.

**Conclusions:**

Our results showed that pikas alter their behavioral (such as FID, the hiding time and the vigilance) response intensity to optimally balance the benefits when exposed to different taxidermy predator species models. We conclude that pikas are able to assess their actual risk of predation and show a threat-sensitive behavioral response.

## Background

It is crucial for prey species to evaluate and respond adaptively to risks posed by their predators, as predators have strong direct and indirect risk effects on prey species. Prey species can be exposed to a wide range of predator species that differ in size [1], density [2], habitat use [3], diel activity [4] and hunting styles [5] in natural systems. Studying the behavioral response intensity of prey to risks posed by different predator species, is therefore an important component of improving our understanding of predator–prey interactions [6, 7].

Predation is a pervasive selection force that influences physiological, morphological, and behavioral adaptations in prey species in order to increase the chances of a successful escape [8]. Generally, the assessment of predation risk is translated into the display of an antipredator behavior. Antipredator behavioral responses to predation risks include a reduction in foraging activity [9, 10], increased vigilance [11, 12], reduced movement [13], reduced use of conspicuous behavioral displays [14], increased hiding time in a refuge or shelter [14, 15], and increased alarm calls [16, 17]. However, these behavioral strategies have associated costs, as they can provoke a reduction in factors such as energy intake, energetic investment in defensive structures, or lower mating success. As risk assessment is difficult to quantify, most studies use Flight Initiation Distance (FID), the hiding time in a refuge and vigilance as the metrics to study the risk levels associated with antipredator behaviors of prey species [7, 14, 18–22]. FID is the distance at which a prey starts to flee upon approach of a predator [23, 24]. Prey approached by predators often flee into refuges and emerge after a brief stay [15, 25]. The hiding time is the time from the moment that prey hides in refuge to the moment that it re-emerges again [26]. Vigilance is the time that prey spend in gathering information that is used to observe predators and assessing the potential predation risk [27]. In general, a longer FID, a longer hiding time in a refuge and higher vigilance means that the prey is experiencing a higher risk of predation [22, 26–33].

A growing number of studies demonstrated that prey can assess their actual risk of predation and shape their antipredator effort accordingly in response to different degrees of predation threat, which supports the threat-sensitive predator avoidance hypothesis. The threat-sensitive predator avoidance hypothesis has been verified in many animals, including insects, crabs, fish, amphibians, reptiles, mammals and birds [23, 28, 34–40]. These studies have shown that prey usually exhibit different anti-predator behavioral response intensities when attacked by predator species which exhibit different levels of predation risks. However, to our knowledge, this hypothesis has rarely been tested in small, burrowing, grassland herbivores in the wild.

The plateau pika (*Ochotona curzoniae*) is a small, diurnal, social burrowing herbivorous lagomorph, which occurs in most areas above an altitude of 3300 m in the Tibetan plateau [41]. The pika is an ideal species to study the assessment of predation risk because they are preyed upon by nearly all of the predators occurring on the plateau. These predators include wolves (*Canis lupis*), Tibetan foxes (*Vulpes ferrilata*), snow leopards (*Uncia uncia*), brown bears (*Ursus arctos*), steppe polecat (*Mustela* *eversmanni*), Alpine weasel (*Mustela altaica pallas*), golden eagles (*Aquila chrysaetos*), upland buzzards (*Buteo hemilasius*), saker falcons (*Falco cherrug*), goshawks (*Accipiter gentilis*), black kites (*Milvus migrans*), little owls (*Athene noctua*) and large-billed crows (*Corvus macrorhynchos tibetosinensis*) [42–44]. Previous studies demonstrated that the Tibetan fox and the Saker falcon are regarded as the most threatening predators for pikas since the Tibetan fox is a pika specialist [45, 46] and pikas are a main food source of the Saker falcon (90% of pellets under the nest of a Saker falcon contained pika remains) [42]. Wolves and crows hunt pikas opportunistically or when other food is scarce, but generally do not pose a serious risk to pikas [7, 47, 48]. In addition, a previous study found that pikas responded differently when they were presented with the calls of different predators [7]. Therefore, it is believed that different types of predators represent different risk levels to pikas [7].

Encounters between predator and prey are rarely observed in nature. For this reason, the predator models have been evaluated using indirect studies [49–53]. In this study, we conducted a field experiment to test ‘the threat-sensitive predator avoidance hypothesis’ using burrowing plateau pikas. We exposed the pikas to four of their common predators, the Tibetan fox, wolf (*Canis lupis*), Saker falcon and large-billed crow, representing different levels of predation risk to the pikas. We assumed that the Tibetan fox and Saker falcon are more threatening predator species than the wolf and large-billed crow based on whether pikas are the main food source for these predators. We hypothesized that the pika would have the ability to assess the level of predation risk and exert different behavior response intensities when exposed to different predator species models. Specifically, we predicted that: (1) pikas would be longer FID when exposed to a more threatening predator species model; (2) the hiding time in a refuge would be longer after an unsuccessful ‘attack’ by a more threatening predator species model; and (3) pikas would allocate more time to vigilance (vigilance is defined as the total duration of time that a pika has its head lifted above its back) when they re-emerge from a refuge after an unsuccessful ‘attack’ by a more threatening predator species model.

## Results

**Fig. 1:**
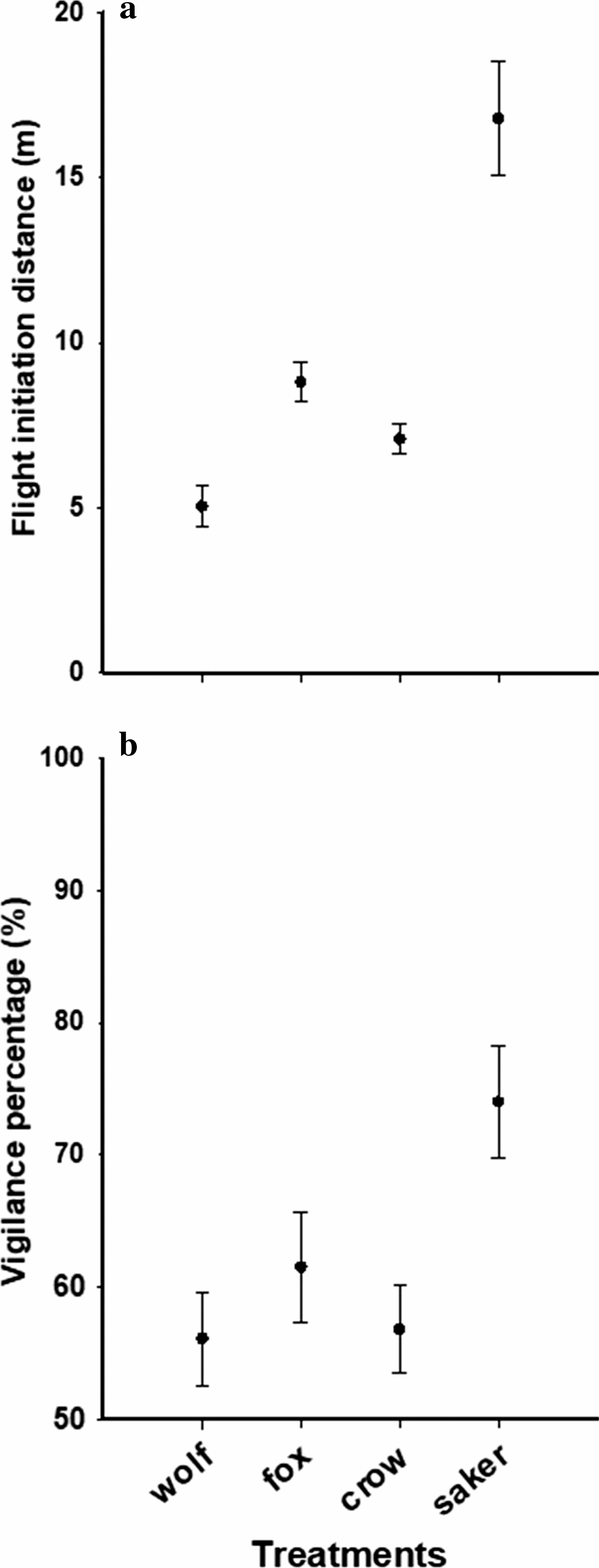
The flight initiation distance (**a**) and the vigilance time (**b**) of pika response to the models of four of their native predators (wolf, fox, crow and saker falcon). Data presented are means with standard errors

**Fig. 2:**
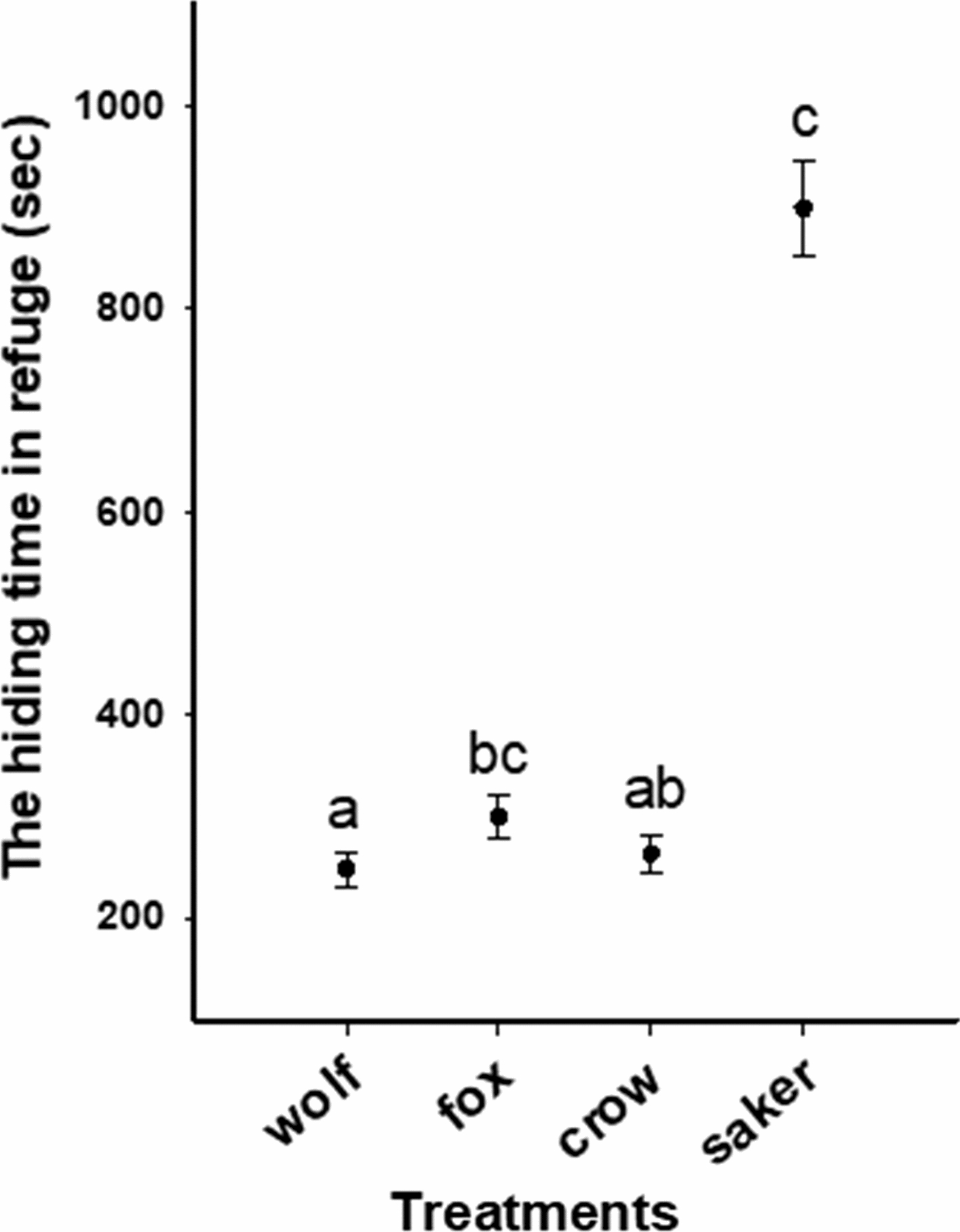
The hiding time of pika response to the models of four of their native predators (wolf, fox, crow and saker falcon). Data presented are means with standard errors. Significant difference (based on a non-parametric multiple test at alpha < 0.05) is denoted by pairs of lower case letters

When approached by a saker falcon, crow, fox or wolf, pikas maintained 16.8 m, 7.1 m, 8.8 m and 5.1 m in FID, respectively (Fig. 1a, b; Fig. 2). Pikas spent 898 s, 263 s, 299 s and 248 s in the refuge, respectively, following an unsuccessful predation by a saker falcon, crow, fox or wolf (Fig. 1a, b; Fig. 2). In addition, when reemerging from the refuge, pikas spent about 74%, 57%, 61% and 56% of their time during the first 10 min on vigilance after an unsuccessful predation by a saker falcon, crow, fox or wolf, respectively (Fig. 1a, b; Fig. 2). A mixed linear model analysis showed that SM (*F *= 7.492, *p *= 0.001) and GS (*F* = 34.864, *p *< 0.001) had significant effects for FID, while P (*F* = 0.058, *p *= 0.944) and EO (*F* = 0.907, *p *= 0.533) had not, and the interaction effects between SM and GS was significant (*F* = 6.187, *p *= 0.002). However, for the hiding time in the refuge, Kruskal–Wallis tests showed a significant difference across different predator species model treatments (*p* < 0.05). After the *p* was adjusted, we found no significant difference between wolf and crow (*p* = 1; Fig. 2), fox and crow (*p* = 0.163; Fig. 2) and between saker falcon and fox (*p* = 0.120; Fig. 2). However, there was a significant difference between wolf and fox (*p* = 0.004; Fig. 2), between wolf and saker falcon (*p* < 0.001; Fig. 2) and between crow and saker falcon (*p* < 0.001; Fig. 2). A mixed linear model analysis showed that SM (*F*-value = 6.329, *p *= 0.002) and GS (*F *= 16.684, *p *< 0.001) had significant effects in vigilance, while P (*F *= 0.780, *p *= 0.468) and EO (*F *= 1.288, *p *= 0.285) had not. However, the interaction effects (*F *= 3.573, *p *= 0.026) of SM and GS did differ significantly in vigilance.

## Discussion

The results from our study provide evidence that pikas display different behavioral response intensities when exposed to different predator species models. The saker falcon is perceived as the greatest threat by pikas as it elicited the strongest anti-predator behavioral response, with the longest FID and hiding time in the refuge, and the highest vigilance percentage. Our results support the ‘threat-sensitive predation avoidance hypothesis’ that pikas have the ability to assess the degree of risk posed by a predator, and that responses are graded in intensity depending on the threat level perceived [49, 54]. Compared to the previously studies [7, 48], this is the first report to assess pika anti-predator behavior in response to the presence of different predator species. These results provide valuable information that may be used in the biological control of one species that can be inhibited by using the interrelationships with another species.

Prey minimizes the cost of escape by remaining where they are until the potential cost of staying outweighs the benefits [19, 21, 55]. This suggests that when a prey detects a predator early, it should delay escape attempts until some criterion relating to escape costs to the cost of not fleeing is met. According to the escape theory, predators with a higher risk are associated with greater FID [56, 57], while FID is expected to be shorter when predation risk is lower [58]. Our results showed that the FID was strongly influenced by the SM. GS is known to affect the ability of prey animals to detect predators [59], which then alter the FID [38]. We also found GS has a significant influence on FID.

Prey often respond to predator attacks by hiding in their refuges or safe microhabitats [60, 61]. However, remaining in refuges can also incur fitness costs, and the decision of when to come out from a refuge after an unsuccessful attack by a predator is an important component of anti-predator behavior [21, 22]. There is a trade-off between staying in refuge with a diminishing risk of predation over time, but with the increased risk of starvation while in the refuge [10, 61, 62]. Cooper and Frederick [21] demonstrated that the hiding time in a refuge should be longer when the perceived risk is higher. Our results are similar to previous studies [24, 63, 64], and support the view that the hiding time in refuge changed with exposure to different predators which present different level of risk.

The level of vigilance is associated with predation risk and vigilance can increase the ability of prey to gather information about the current predation risk [7, 9]. In addition, the vigilance level of prey depends on the level of previous predation risk [9]. In general, prey reduced foraging time and engaged in anti-predator behavior when the previous predation risk was high [9]. Our results indicate that the vigilance level was significantly higher in response to a saker falcon compared to the other predators, which indicates pikas perceive the saker falcon as the greatest risk of our four test predator species.

Aerobic movements of animals is energetically costly, especially in QTP [41]. The reduction of unnecessary aerobic movements lowers energetic costs and can increase the survival rate of pikas [41]. Pikas have adapted to display varying anti-predator behavioural response intensities depending on the level of risk posed by different predators [7]. The results of the present study indicate that the saker falcon is regarded as the most dangerous predator because pikas elicited the strongest anti-predator response (for example, the furthest FID, the longest hiding time in refuge and the highest vigilance percentage) when exposed to it. A possible explanation for the difference in responses elicited by the different predators is the difference in the approach speed of the different predator species. Zhang et al. [7] suggested that raptors (eagle and falcon) are more threatening than beasts (fox and wolf) because raptors approach faster. In contrast, our results indicate that the threat of a fox is greater than that of a crow [7]. Thus, a more likely explanation for the difference in behavioral response intensities are related to whether the pika is the main food resource for the specific predator. In addition, our results also indicate that the saker falcon poses a greater threat to pikas than the fox, implying that pikas are able to evaluate risk levels by assessing the predator visually and having stronger antipredator behavioral responses when facing a more threatening predator. The ability to discriminate between more and less dangerous predators can have significant advantages for pika survival. Many other animals also vary their behavioral response intensity depending on the predator species [23, 28, 34–40], and this adaptation is as a result of co-evolution with predators over millions of years [7]. However, it is not known whether the ability of pika to discriminate between predators is innate or based on experience and would require further studies to elucidate this.

Predators play an important role in the control of pikas as the direct and indirect predation risk effects can significantly affect the fertility and survival of pikas [45, 65]. Over the past decades, plateau pikas have been targeted for control by poisoning primarily because they are believed to have a negative impact on rangeland and compete with livestock for food [43]. An unfortunate consequence of these poisoning campaigns to kill pikas is that the predator species may inadvertently be poisoned [43]. Besides that, many predators of pikas are being killed for profits [48]. The situation is further exasperated by the fact that the pika fertility is far greater than that of its predators [48], and the pika population can recover rapidly to its original state in a short time [66]. whereas the predator numbers remain low due to the killing and poisoning campaigns. Essentially the natural mechanism of pika population control is eliminated from the system, and the pika populations continue to increase unchecked. Therefore, it is imperative that the poisoning campaigns and the killing of carnivore campaigns should be halted.

## Conclusions

Our results show that pikas are able to discriminate between predator species which present different levels of risk and alter their response according to what is likely to be a larger threat. In addition, our results also provide support to previous studies suggesting that the saker falcon is the most threatening predator of pikas in the alpine meadow of the Qinghai-Tibet Plateau. Finally, given the critically important role of predators of pikas in controlling their population densities we urge that the campaigns to poison pikas and the killing of their carnivore predators should be terminated.

## Materials and methods

### Study site

The study site is located in a natural alpine meadow in Luqu County, Gansu province, northwestern China. Geographically, the study site is located on eastern part of Qinghai-Tibetan plateau (lat. 34° 14′ N; long. 102° 13′ E; alt. 3650 m). The site has a typical alpine continental climate, with mean annual temperatures of 2.3 °C. The average annual precipitation is 543.6 mm, and occurs predominantly between June and September. The main soil type is subalpine meadow soil. The vegetation type is alpine meadow, and dominant species is *Kobresia humilis*, *Elymus nutans, Festuca ovina L, Polygonum viviparum L, Anemone obtusiloba D.* The inclination of study site (plateau pika habitat) is about 13° on a western slope. In this area, the distribution of pika families is patchy and each family contains 4–7 individuals. In our study area, the range of the active area of a pika family is about 470–680 m^2^.

### Experiment design

**Fig. 3:**
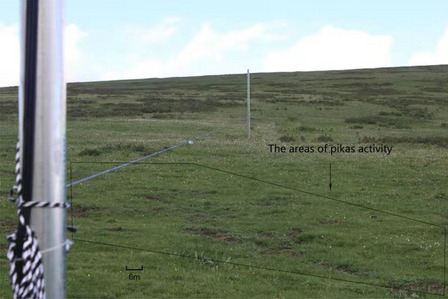
The Sample selection and the black wireframe is the active area of pikas. The range of active area of a pika family is about 470–680 m^2^ in our study area

**Fig. 4:**
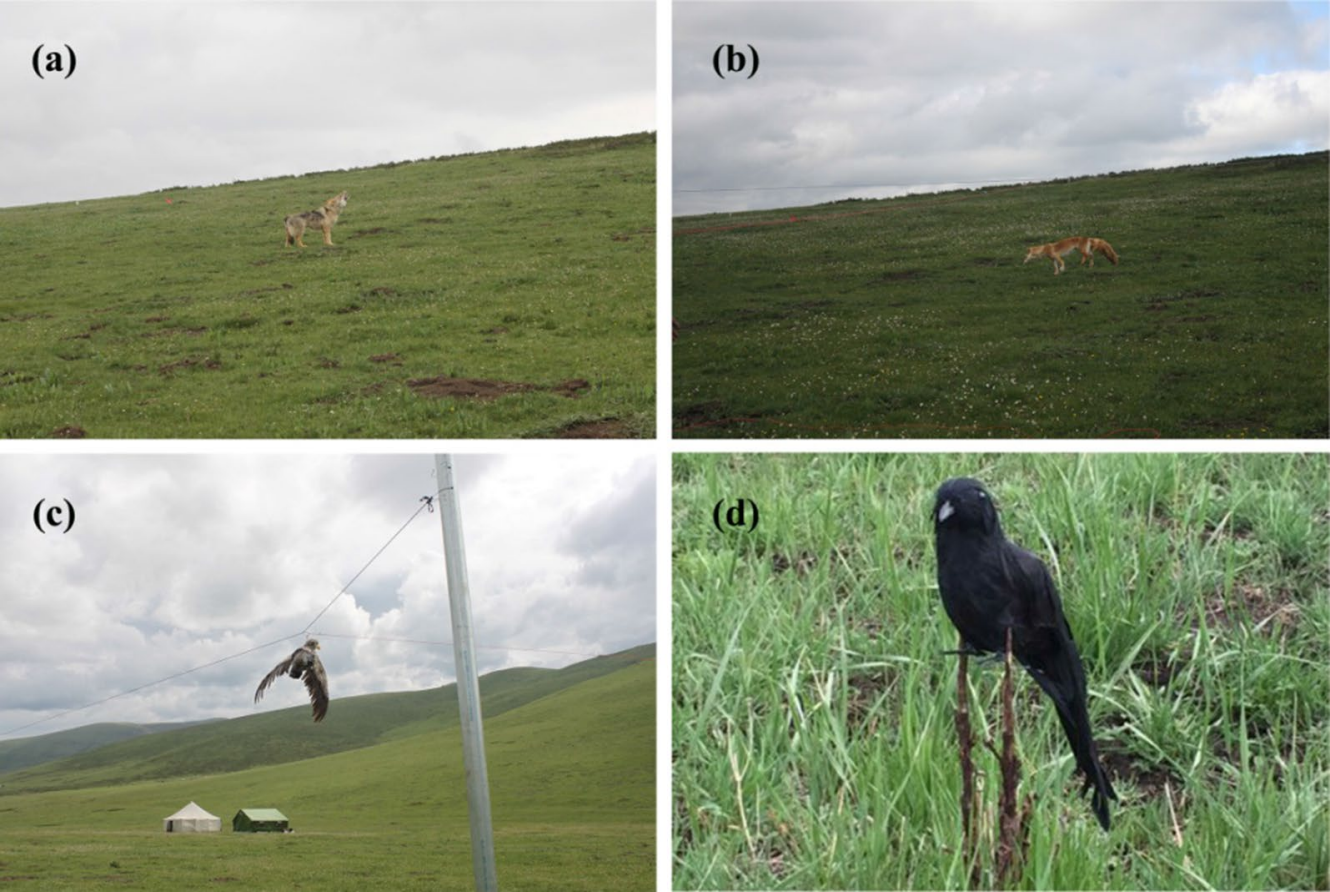
Four different taxidermy predator species models: **a** Tibetan fox (*Vulpes ferrilata*). **b** Wolf (*Canis lupis*). **c** Saker falcon (*Falco cherrug*). **d** Large-billed Crow (*Corvus macrorhynchos*)

The experiments were conducted 15–29 June, 2016, after the breeding season. We randomly selected three different pika populations (P) which were spatially non-adjacently distributed in our study site. Ten days before the start of the experiment, we placed two iron pillars (50 cm diameter, 3 m high) in each area, where one pillar was situated in the pika colony, the other was situated on the slope above the pika habitat, and the distance between the two pillars was 50 m (Fig. 3). The two pillars were connected by a rope that was strong enough to hold and slide the predator models. The height of the rope was adjusted depending on the predator species. We fixed an infrared high definition camera (Huian: WL-1008T, LED, 2megapixel, 12.8, Progressive ScanCMOS, 1920 × 1080 fps) that can rotate 360° on the pillar that was in the colony, and used a cable to connect it to a computer (Lenovo, G5050) in a tent that was 400 m away from the pika colony. During the experiments, the anti-predator behavior of the pikas were observed and recorded. We tested four different conditions: a wolf (length: 135 cm, width: 25 cm, height: 30 cm), a Tibetan fox (length: 50 cm, width: 15 cm, height: 35 cm), a large-billed crow (length: 10 cm, width: 5 cm, height: 15 cm) and a saker falcon (length: 45 cm, width: 150 cm, height: 25 cm). The four predator models served as the predator species models (SM) (Fig. 4). Each population was tested for 4 cycles (each cycle was 2 days long) and the interval between cycles was at least 2 days. A cycle consisted of presenting each of the four predators to a colony of pikas. The order (EO) of the predators was randomized to avoid habituation of the pikas to the experimental procedure, while the interval between different predators in a cycle was at least 3 h. In addition, we recorded the survey dates (SD) of SM in different P.

During the experimental procedures, the predator models were placed on the rope and a person dragged the model from the upper pole to the lower pole inside the pika colony with a rope by walking 80 m away (Human activities affect the activity of pikas at distances closer than 30 m) [66], parallel to the model at a speed of 5 m/s. When pikas hid in their burrows, the predator model was moved back up to the upper pole. Tests were conducted in the morning during peak hours of pika activity (8:00–9:00) on a sunny day. Taking into account the height of the animal and its hunting style, we adjusted the height to 40 cm, 90 cm, 120 cm and 130 cm for the tibetan fox, wolf, large-billed crow and Saker falcon, respectively. Trials were stopped if there were predators in the surrounding environments.

We analyzed the videos at one quarter speed and scored the hiding time and vigilance using J Watcher 1.5.0. In our experiments, we only observed adult pikas whose vigilance direction was opposite to that of the approaching predator model to determine the FID because vigilance direction can influence the FID [23, 67]. In addition, group size (GS) was quantified as it can also influence FID [7]. When all experiments were analyzed, we measured the FID and the refuge distance (RD) measured for individual observed pikas, the FID and refuge distance was measured to the nearest 0.1 m. The hiding time was defined as the period between first adult retreating, to the first adult pika emerging again from burrows [7]. Finally, we measured the vigilance percentage within ten minutes once the pika has left the burrow entrance. The vigilance is the total duration of time that a pika has its head lifted above its back, regardless whether it was quadrupedal or bipedal [68].

### Data analyses

To improve normality, the FID was reciprocally transformed and vigilance was square root transformed, and were tested with general linear models in SPSS 22.0. Pearson correlation coefficients were used to identify collinearity among independent variables. To control for multicollinearity, we tested correlations of pairs of independent variables. Association between variables was assessed using the Spearman correlation index (Rs) and was considered significant when p < 0.05. We only maintain one of the correlated collinear variables in the next analysis. The effect of SM on the FID was analyzed using a mixed linear model with GS and RD as covariates, P and SD and EO as random variables and SM as a fixed variable, RD and SD were not included as predictors in the LMMs as GS and RD, SD and EO were highly collinear. Then we fit a model without RD and SD to test for the main effects. The effect of SM on the vigilance was analyzed using a mixed linear model with GS as covariates, P and SD and EO as random variables and SM as a fixed variable, SD was not included as a predictor in the LMMs as SD and EO were highly collinear. Then we fit a model without SD to test for the main effects. All interactions among these were included in the model and removed if not significant. However, hiding time was not normally distributed despite multiple transformations, therefore we used Nonparametric Tests (Kruskal–Wallis) followed by all pairwise multiple comparisons.

## Data Availability

The datasets used and analysed during the current study are available from the corresponding author on reasonable request.

## Abbreviations

FID: Flight initiation distance
P: Pika populations
SM: Species models
EO: Order
SD: Survey dates
GS: Group size
